# Distinct patterns in neuromuscular adaptation to repeated perturbations in chronic ankle instability

**DOI:** 10.1186/s12984-025-01838-y

**Published:** 2025-12-12

**Authors:** Xiaohan Xu, Joanna Bowtell, William R. Young, Daniel T. P. Fong, Genevieve K. R. Williams

**Affiliations:** 1 Public Health and Sports Sciences Department, University of Exeter, Exeter, EX1 2LU UK; 2 National Centre for Sport and Exercise Medicine, School of Sport, Exercise and Health Sciences, Loughborough University, Loughborough, LE11 3TT UK

**Keywords:** Chronic ankle instability, Muscle co-contraction, Neuromuscular adaptation, Postural control, Perturbation

## Abstract

**Background:**

Neuromuscular impairments following ankle sprains are central to chronic ankle instability (CAI), potentially leading to compensatory muscle co-contraction to regulate ankle stiffness, particularly in response to external perturbations. The acute effect of practice on muscle activation and postural responses reveal initial deficits in motor control and indicate the capacity of individuals with CAI to overcome these deficits within their specific constraints. This study aimed to examine adaptive changes in lower limb muscle co-contraction between CAI and healthy control (HC) participants during repeated perturbations and assess whether practice influences postural control and strategy.

**Methods:**

Twenty-three CAI and 23 HC participants performed a single-leg balance task involving repetitive mediolateral perturbations. Surface electromyography, ground reaction force and whole-body kinematics were recorded, and margin of stability (MoS) and the coupling between MoS and ankle-/ hip-joint torques were calculated.

**Results:**

Individuals with CAI demonstrated different adaptive changes in muscle co-contraction for Tibialis Anterior (TA) - Peroneus Longus (PL) and TA - Soleus compared to HC. In healthy controls, TA-PL co-contraction decreased significantly with practice, while no changes were observed in the CAI group. Repeated perturbations improved MoS and MoS-Hip torque coordination in CAI, suggesting improved postural control and hip strategy.

**Conclusions:**

Rehabilitation for CAI should target both the whole-body coordination and ankle adaptation exercise since ankle joint appears refractory to change in CAI individuals. Future research should explore whether co-contraction patterns influence risk of ankle sprain in CAI populations, linking lab-based performance to real-world injury risks.

**Supplementary Information:**

The online version contains supplementary material available at 10.1186/s12984-025-01838-y.

## Introduction

Chronic ankle instability (CAI) develops in approximately 50% of individuals following a lateral ankle sprain [1], which in itself is one of the most common musculoskeletal injuries [2], affecting around 70% of the population [3]. CAI describes the chronic ankle symptoms, such as ongoing pain, giving way and instability [4], which impairs sensorimotor function, postural control and increases the likelihood of recurrent injuries [5].

Individuals with CAI often exhibit postural control deficits and altered movement patterns during unilateral weight-bearing tasks, such as single-leg stance [6, 7], landing [8], and lateral stepping down [9, 10]. However, the mechanisms of these postural control deficits, particularly relating to neuromuscular control are not well understood for unilateral tasks. During dynamic balance, the margin of stability (MoS) defines mechanical stability, based on the distance between the extrapolated centre of mass (XCoM) and the base of support (BoS) [11]. MoS characteristics are task- and population- dependant. Greater MoS (i.e. absolute distance between XCoM to BoS) reflects a less conservative, and/or more unstable approach to maintaining balance. This pattern has been observed in various pathological conditions, such as stroke [12, 13], amputation [14], and multiple sclerosis [15], and associated with instability under physically demanding tasks, such as dual-task walking [16], perturbation-induced gait [17], and walk with higher stride frequencies [18]. In contrast, a decreased MoS indicates a more vigilant, conservative strategy that enhances mechanical stability, commonly reported in pathological gait, such as in Parkinson’s disease [19, 20] and in spinal cord injury during slow walking [21] when compared to healthy controls. Recent studies have revealed aging effects on joint contributions to postural control by assessing the temporal coupling of joint motion or torque with MoS in older and young healthy adults during walking [22, 23]. To the best of our knowledge, it remains unclear whether CAI individuals adopt a more unstable or conservative strategy during perturbed balance, and how joint torque contribute to postural control. Uncovering compensatory mechanisms used by CAI individuals will inform future research linking balance strategies to recurrent injury risk, and guide rehabilitation approaches prioritising either enhancing postural stability or adaptability in the strategy used.

Muscle co-contraction is essential for joint stabilisation during balance control. It involves activating antagonist muscle pairs to generate opposing torques at the joint [24]. Specifically, lower limb muscle co-contraction contributes to joint stiffness when the intrinsic stiffness provided by muscle-tendon units and surrounding tissues is insufficient for maintaining upright posture [25], leading to greater resistance to external forces. Increased co-contraction of the lower limbs during walking and balance has been found in older compared to younger adults [26]. However, excessive co-contraction might lead to greater energy expenditure during balance and locomotion [27], and further impair reactive balance [28] and increase the risks of falls [29–31]. A reduction in co-contraction has been shown to be an adaptive change induced by repeated perturbations, suggesting improved efficiency with training [32, 33].

Individuals with CAI often have increased talocrural joint laxity [34], and as a results potentially use compensatory muscle co-contraction to regulate ankle stiffness. However, findings on activation patterns of the ankle muscles in CAI population are mixed. For example, [35] found a greater Tibialis Anterior-Peroneus Longus (TA-PL) co-contraction in the CAI group compared to a control group during the eyes-closed single-leg balance. For TA-PL co-contraction during single-leg landing, CAI individuals exhibited either no difference [36, 37], increased [38] or decreased co-contraction [39], compared to healthy controls. Additionally, lower TA-peroneal co-contraction was reported in CAI than healthy control during medium latency responses to standing inversion perturbations [40]. In individuals with CAI, increased co-contraction can serve as a functional compensation for increased talocrural joint laxity, but excessive co-contraction is dysfunctional and represents less adaptive motor control. However, inconsistencies in previous research have left the mechanisms underlying co-contraction unclear. Given the co-contraction pattern could be shaped by repeated perturbations [32, 33], the current study will apply repeated sinusoidal perturbations to challenge both agonist and antagonist muscle and increase co-contractions, in order to compare muscle co-contraction control strategy in CAI versus healthy control population.

We will document acute changes in muscle activation (i.e. muscle co-contraction) and postural control (i.e. MoS, MoS-joint torque coupling) in response to repeated perturbations. These immediate adaptations can provide insight into motor control deficits and indicate the capacity of individuals with CAI to overcome these deficits within their specific constraints. For instance, balance training seems to improve balance and self-perceived ankle function, but effectiveness in improving sensorimotor deficits [41] and muscle inhibition [42] is questionable. Investigating acute strategic changes during perturbation-based training could clarify initial sensorimotor control, potentially informing long-term motor skill development in CAI [43]. The primary aim of the current study was to investigate differences in adaptive changes in balance performance and lower limb muscle co-contraction between CAI and healthy control (HC) during repetitive perturbation trials. We hypothesised that both groups would decrease muscle co-contraction with practice [33]. The second aim was to assess whether repeated perturbations could improve postural control in CAI individuals, focusing on the margin of stability (MoS) and coordination between MoS and hip/ankle joint torque. The temporal coupling of MoS with ankle, hip, and trunk torques indicates how effectively joint actions generate optimal stabilisation with minimal energy expenditure [23], in our case, in response to BoS movements elicited by moving platform [44]. Since higher temporal coupling between MoS and joint torques reflects more effective joint actions to external perturbations, we hypothesised that CAI group would show decreased MoS and increased MoS-Hip coordination with repeated perturbations.

## Methods

### Participants

Twenty-three participants with CAI and 23 healthy controls (HC) took part in the study after providing written informed consent. Ethical approval was gained a priori. The Sports and Health Sciences Department Ethics Committee approved the study (1071581). All methods were performed in accordance with the Declaration of Helsinki (2013) and the University of Exeter Ethics Policy ([45]).

Participants with CAI were selected based on the International Ankle Consortium guidelines [4]. Inclusion required a history of a lateral ankle sprain occurring at least 12 months prior, with a most recent sprain more than 3 months prior, and at least 2 episodes of giving way or recurrent sprain in the 6 months prior to study enrolment. Impaired ankle function was confirmed by a Cumberland Ankle Instability Tool (CAIT) score of less than 24 [46]. Control group participants had no history of lateral ankle sprains. All participants were aged 18–35 years, with no acute musculoskeletal injuries in the three months prior to testing. Exclusion criteria included visual or hearing impairments, dizziness, recurrent falls, vestibular dysfunction, lower extremity fractures, pain, surgery, or prior professional balance training experience. One participant in the CAI group did not complete enough successful trials during the practice blocks and was excluded. Thus, data analysis included 22 CAI and 23 HC participants. The participant characteristics were similar between groups: sex, age, height, body mass, and physical activity level, assessed using the International Physical Activity Questionnaire-Short Form (IPAQ-SF) (Table 1).

**Table 1:** Participants’ demographics and anthropometrics

	CAI (*n* = 22)	HC (*n* = 23)
Sex	Female − 10	Female − 9
	Male − 12	Male − 14
Age, years - mean ± SD	23.0 ± 3.2	24.5 ± 3.9
Height, m - mean ± SD	1.72 ± 0.09	1.72 ± 0.08
Body Mass, kg - mean ± SD	70.4 ± 9.3	65.2 ± 8.5
IPAQ-SF	Moderate − 12	Moderate − 12
	High − 10	High − 11
CAIT Score	17.6 ± 4.1	30.0 ± 0

*SD* standard deviation, *IPAQ-SF* International Physical Activity Questionnaire-Short Form, *CAIT* Cumberland Ankle Instability Tool

### Study procedures

**Fig. 1:**
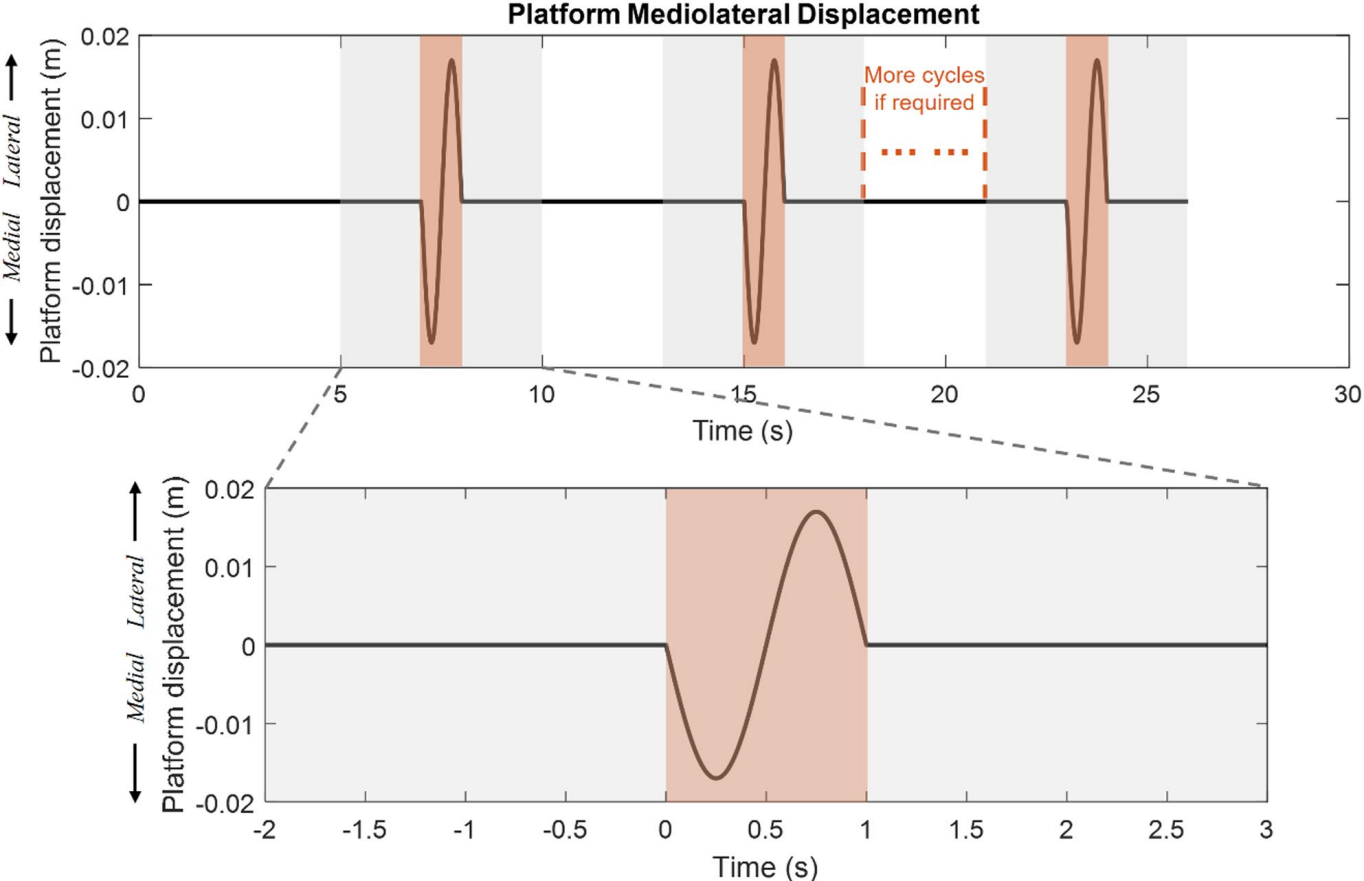
Platform motion during block two and three. Each 1-second mediolateral sinusoidal perturbation phase (shaded in orange) is followed by a 7-second recovery phase for preparation and recovery. Platform moves with a 0.017-m amplitude, starting with a medial displacement. Trials requiring participants to maintain successful balance for 5 s are shaded in grey, indicated in the lower panel. Additional cycles were added if needed to achieve three successful trials

The test was conducted over three blocks, each separated by at least a 5-min seated rest break to minimise muscular fatigue during the single-day visit. The first block aimed to achieve the first successful trial. Block two and three were intensive practice session, using the same perturbation parameter as in block one, requiring participants to maintain single-leg stance and balance during intermittent perturbations until they achieved three successful trials. The platform motion consisted of a 1-second, 1.7-cm amplitude, 1-Hz mediolateral sinusoidal movement, followed by 7-s of stable standing for recovery and preparation (Fig. 1). During the 7-second recovery period, participants were instructed to stand still on one leg. A 7-second interval was chosen as it was sufficient for participants to recover from a failed trial, as confirmed through pilot testing, and allowed for consistent delivery of repetitive perturbations to facilitate intensive practice. A failed trial was defined as losing balance during 5-s trial (2-s baseline, 1-s perturbation, 2-s recovery), the lifted leg touching down, taking additional steps, jumping, or creating frictional movements against the platform surface, as well as visually apparent movements of the lifted legs and arms beyond minor adjustments.

During the tests, participants performed barefoot single-leg balance tests on the affected side in the CAI group. For those with bilateral ankle sprains, the side with the lower CAIT score was selected. Height-, gender-, and IPAQ-SF-matched HCs were tested on the corresponding side, and we assumed a similar distribution of leg dominance across both groups. While leg dominance may influence balance strategies, a systematic review suggests the effect is not significant [47]. Participants were instructed to maintain balance in the standardised single-leg stance as described in the previous study [7], hands on waist, facing the anteroposterior axis of the global coordinate system, ensuring their standing foot was pointed straight ahead. They were instructed to maintain their gaze on a target positioned at eye level, approximately three meters away on a wall.

### Data collection

Participants performed the balance task on a 3.6 m × 3.6 m instrumented moveable floor in the Exeter VSimulators facility, as described in the previous study [7], using the same setup and equipment. This included a 24-camera motion capture system (OptiTrack, NaturalPoint Inc., Corvallis, OR, USA, 100 Hz) with 57 reflective markers based on the Biomech-57 marker set (OptiTrack 48, AMTI force plates (120 cm × 120 cm, Advanced Mechanical Technology Inc., 1 kHz).

Activation of the Tibialis Anterior (TA), Peroneus Longus (PL), and Soleus (SOL) muscles was recorded using wireless surface electromyography (EMG) electrodes (37 × 27 × 13 mm; Trigno, DELSYS, Boston, MA, USA) at a sampling rate of 1925.925 Hz, processed through Trigno Discover software. An EMG with an integrated accelerometer (sampled at 370.3704 Hz) was placed on the platform to synchronise the platform motion with the EMG channels. The accelerometer signal was resampled to 1925.925 Hz using interpolation for time alignment, and the peak acceleration was used to identify the onset of platform motion. EMG electrode placement followed SENIAM guidelines [49]. Skin preparation included abrasion with Nuprep^®^ Gel (Weaver and Company, US) and cleaning with alcohol to optimise signal quality. Baseline noise was assessed during setup, ensuring a root mean square (RMS) value below 15 µV and a signal-to-noise ratio (SNR) above 10 [50]. SNR was calculated using the formula: SNR = 20*log (RMS of signal during muscle contraction/RMS of baseline noise) by asking participants to contract their muscles. EMG signals were amplified during a bio-amplifier (Trigno Wireless System) with a bandwidth of 20–450 Hz.

The raw EMG data were firstly filtered by using a 4th -order Butterworth band pass filter (10–400 Hz), followed by full-wave rectification. A low-pass a 4th -order Butterworth filter at 20 Hz was then applied to obtain the linear envelope. The EMG for each muscle was normalised as a percentage of the maximum EMG value recorded across all successful perturbation trials. Muscle co-contraction was calculated for two muscle pairs: TA-PL (inversion-eversion) and TA-SOL (dorsiflexion-plantarflexion). The co-contraction index (CCI) was quantified over the 1-second perturbation period based on [51]’ s method as follows:

$$ \begin{aligned} \:{\mathrm{CCI}}\: = \: & 2 \times \:\sum \: _{{i = 1}}^{n} \frac{{min\left[ {{\mathrm{EMG}}_{{{\mathrm{AG}}}} \left( i \right),{\mathrm{EMG}}_{{{\mathrm{ANT}}}} \left( i \right)} \right]}}{{{\mathrm{EMG}}_{{{\mathrm{AG}}}} \left( i \right) + {\mathrm{EMG}}_{{{\mathrm{ANT}}}} \left( i \right)}} \\ & \times \:100\% \\ \end{aligned} $$

where *min* denotes the minimum value between the agonist (EMG_AG_) and antagonist (EMG_ANT_) muscles at each time point $\:i$. The parameter $\:n$ represents the total number of data points in the time window. CCI values for TA-SOL and TA-PL were used for further analysis. The base of support (BoS) was defined by the instantaneous centre of pressure (CoP) position for a more accurate representation of the effective BoS[11, 52]. The centre of mass (CoM), joint torques, and margin of stability (MoS) were calculated based on [53], [54] and [11]. Data from the 1-second perturbation phases were extracted for analysis (Fig. 1), which included one trial from block one and three trials each from practice session 2 and 3. All the data were processed in MATLAB 2022b (MathWorks, Inc., Natick, Massachusetts).

### Statistical analysis

Data from the first three successful trials in block two and three were averaged to represent each participant’s performance in that block in statistical analysis. To identify differences in MoS, ankle and hip torque in time-series between groups during motor adaptation induced by repeated perturbations, a two-way repeated measures ANOVA was conducted with 2 groups (CAI and HC) × 3 sessions by using the SPM1D package (v.0.4.11, available at https://spm1d.org) in MATLAB. Since the current version of SPM1D (v.0.4.11) does not support unbalanced design (22 CAI vs. 23 HC), one participant from HC group was excluded to create a balanced dataset for time-series analysis, with a leave-one-out sensitivity analysis confirming robustness to this exclusion. Session main effects were further analysed within each group using SPM1D one-way repeated measures ANOVA.

Cross-correlation was used to quantify the relationship between MoS and joint torque waveforms at zero-phase lag across two time-windows (0–0.5 s, 0.5–1 s) based on pre-defined postural responses. For each participant, the absolute values of cross-correlation at zero-lag were calculated, with strength categorised as strong (0.5–1), moderate (0.3–0.5), or weak (0–0.3). Once the normal distribution of data was confirmed, the two-way repeated ANOVA were conducted to evaluate motor adaptation effects on groups in cross-correlation coefficients (i.e. hip-pairing and ankle-pairing) and muscle co-contraction (CCI_TA−PL_, CCI_TA−SOL_) by using SPSS (IBM SPSS 29, NY: IBM Corp). Bonferroni post hoc tests were computed. Partial Eta Squared effect sizes were used to determine the magnitude of differences and were interpreted as small (0.01–0.06), moderate (> 0.06–0.14), and large (> 0.14) [55]. Pearson’s correlation analyses were conducted in the CAI group to examine the relationships between ankle function by CAIT scores and the overall average CCI TA-PL and CCI TA-SOL across the three practice blocks. The statistical significance was set at *p* < 0.05.

## Results

There were no significant group differences in the number of attempts to achieve the first successful trial during block one (CAI: 3.7 ± 2.4; HC: 2.8 ± 1.1; Z = −0.955, *p* = 0.340, U = 213) or the total number of perturbation repetitions, including successful and unsuccessful attempts across practice blocks two and three (CAI: 12.5 ± 3.1; HC: 11.1 ± 2.0; Z = −0.121, *p* = 0.228, U = 200.5), as assessed by the Mann-Whitney U test. All participants completed three successful trials within seven attempts during practice block two and three.

### Margin of stability

**Fig. 2:**
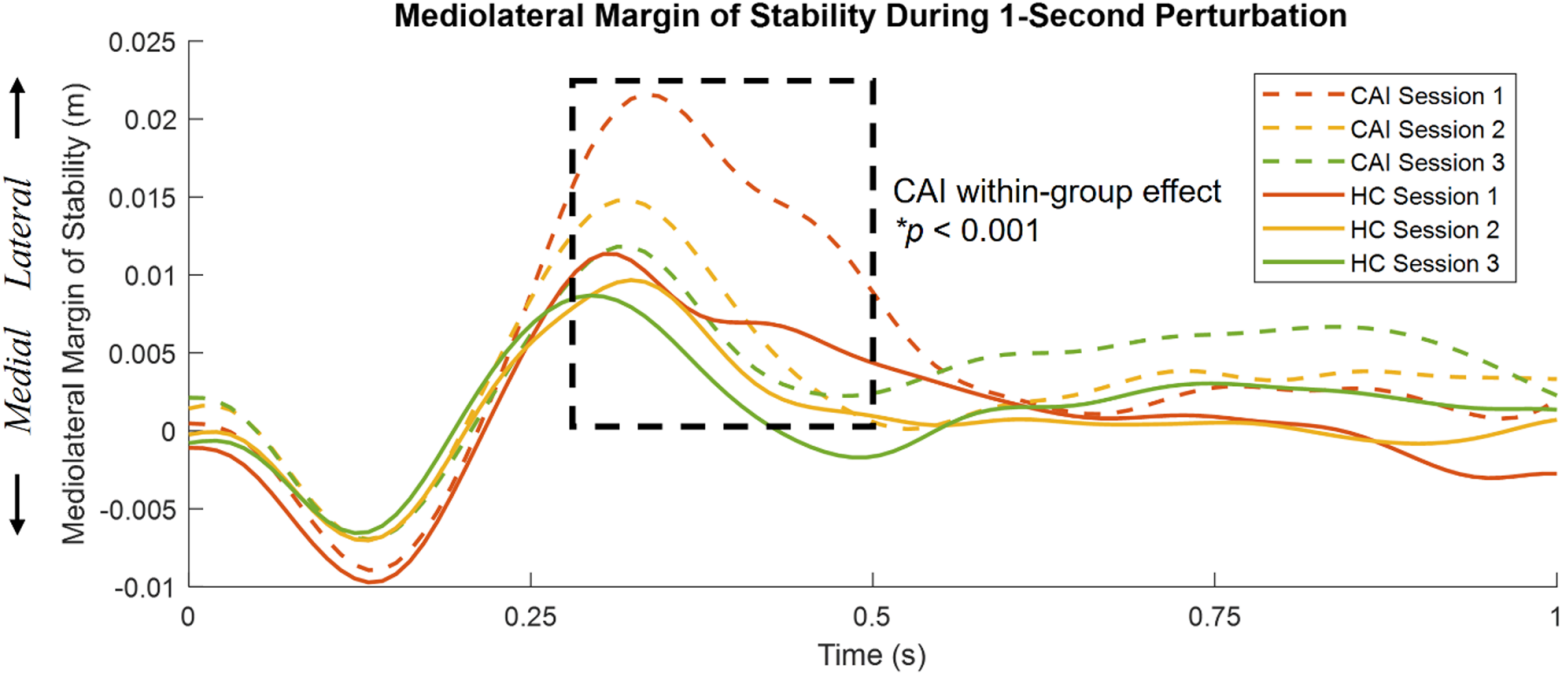
Mean mediolateral margin of stability (MoS) during the 1-second perturbation for CAI (dashed lines) and HC (solid lines) across three sessions. Significant within-group differences among sessions in the CAI group are highlighted by the dashed square (*p < 0.001). No significant within-group differences in the HC group across three sessions. Statistical analysis was conducted by SPM1D one-way repeated measures ANOVA

There was no interaction effect on MoS, suggesting that motor adaptation effects on MoS occurred independently of group differences (Figure S1). There was group main effect (F^*^_(1,42)_ = 9.214, *p* = 0.05), suggesting CAI exhibited greater MoS than HC during 0.35 s regardless of sessions. There was main effect of session during 0.18 s (F^*^_(2,84)_ = 5.9, *p* = 0.05) and 0.3–0.5 s (*p* < 0.001) regardless of groups, indicating the distance between XCoM and BoS was closer with repetition. For the CAI group, between session effects showed a significant decrease in MoS as perturbation repeated during 0.29–0.5 s (Fig. 2), when the platform motion changes from medial to lateral (F^*^_(2,42)_ = 6.553, *p* < 0.001). This suggests the follow-up practice during session two and three improved the effectiveness of postural control responses to platform transitions.

### Joint torque and cross-correlation

**Fig. 3:**
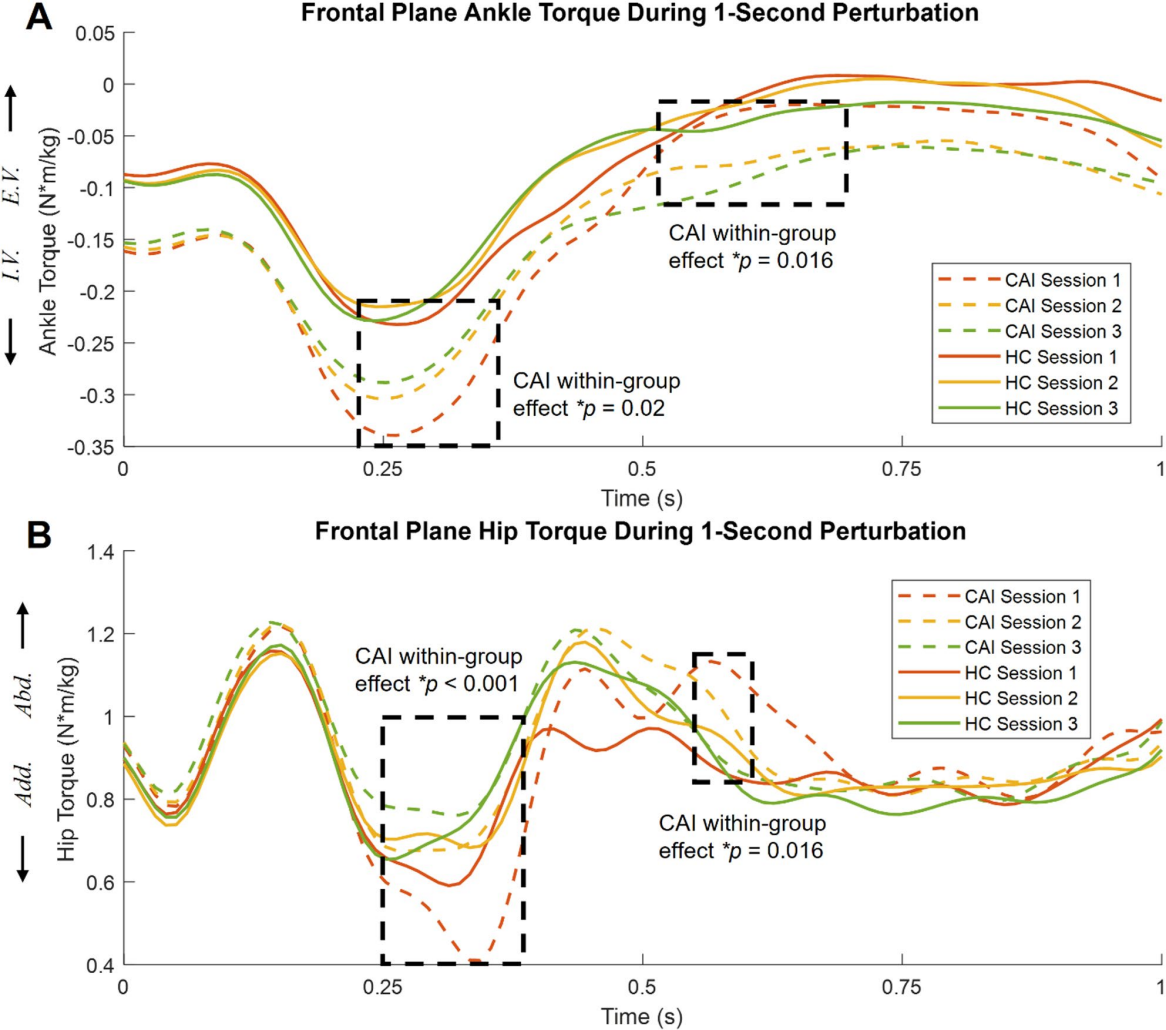
**A** Mean frontal plane ankle torque during the 1-second perturbation for CAI (dashed lines) and HC (solid lines) across three sessions. Significant within-group differences among sessions in the CAI group are highlighted by the dashed square during 0.24–0.35 s and 0.54–0.67 s. No significant within-group differences in the HC group across three sessions. **B** Mean frontal plane hip torque during the 1-second perturbation for two groups. Significant within-group differences among sessions in the CAI group are highlighted by the dashed square during 0.25–0.39 s and 0.58–0.61 s. No significant within-group differences in the HC group across three sessions. Statistical analysis was conducted by SPM1D one-way repeated measures ANOVA

No interaction effect was observed for frontal plane ankle torque, suggesting the effects of practice on ankle torque were independent of group differences (Figure S1). A significant group main effect (F^*^_(1,42)_ = 8.494, *p* = 0.014) was found during 0.18–0.33 s, suggesting a greater invertor torque in the CAI than HC group. A main effect of session (F^*^_(2,84)_ = 5.532, *p* < 0.05) indicated that participants decreased their invertor torque during 0.28–0.33 s and evertor torque during 0.57–0.73 s and 0.9–0.95 s as sessions progressed, regardless of group. Within the CAI group, there was a significant reduction in ankle invertor torque during 0.24–0.35 s and reduction in evertor torque during 0.54–0.67 s as perturbation repeated (F^*^_(2,42)_ = 5.958, *p* < 0.05, Fig. 3A).

Interaction effects were observed for hip torque (F^*^_(2,84)_ = 6.809, *p* = 0.039) during 0.36–0.37 s (Figure S1). Specifically, the HC group showed no significant changes, while the CAI group exhibited decreased adductor torque during 0.25–0.39 s and decreased abductor torque during 0.58–0.61 s (Fig. 3B).

**Fig. 4:**
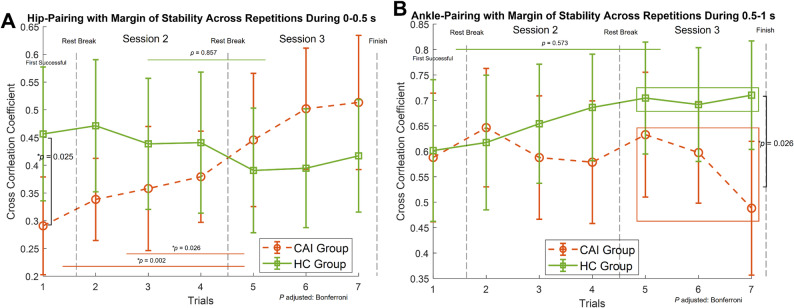
**A** Mean absolute cross-correlation coefficient (± 95% CI) between Margin of Stability (MoS) and hip torque during 0–0.5 s for CAI (dashed lines) and HC (solid lines) across seven successful trials in three sessions. Vertical dashed lines indicate session boundaries, each separated by a at least 5-minute seated rest. Trials 2–4 and 5–7 were averaged for statistical analysis. Post hoc comparisons revealed group differences during the first successful trial (*p* = 0.025). Within the CAI group, session 3 showed significantly higher coefficients compared to session 1 (*p* = 0.002) and session 2 (*p* = 0.026). **B** Mean absolute cross-correlation coefficient (± 95% CI) between MoS and ankle torque during 0.5–1 s for CAI and HC groups. No significant changes were observed within groups across repetitions. A significant between-group difference was identified in session 3 (*p* = 0.026)

Cross-correlation values between MoS and hip/ankle joint torques were in the range of 0.3–0.7 across three sessions, suggesting a moderate to strong relationship (Table 2). Statistical analysis suggests a significant interaction effect for the MoS-hip torque pairing during 0–0.5 s (F _(2,86)_ = 6.722, *p* = 0.002). Post hoc pairwise comparisons revealed distinct patterns of change across sessions for each group (Fig. 4A). The CAI group showed an increased cross-correlation coefficient over the three sessions, indicating improved dynamic postural coordination with the hip, while no significant changes were observed in the HC group. No interaction effects were observed for postural coordination with hip or ankle torque during 0.5–1 s. However, during session three, the CAI group exhibited a significantly lower ankle-MoS pairing compared to the HC group (*p* = 0.026, Fig. 4B).

### Muscle co-contraction

**Fig. 5:**
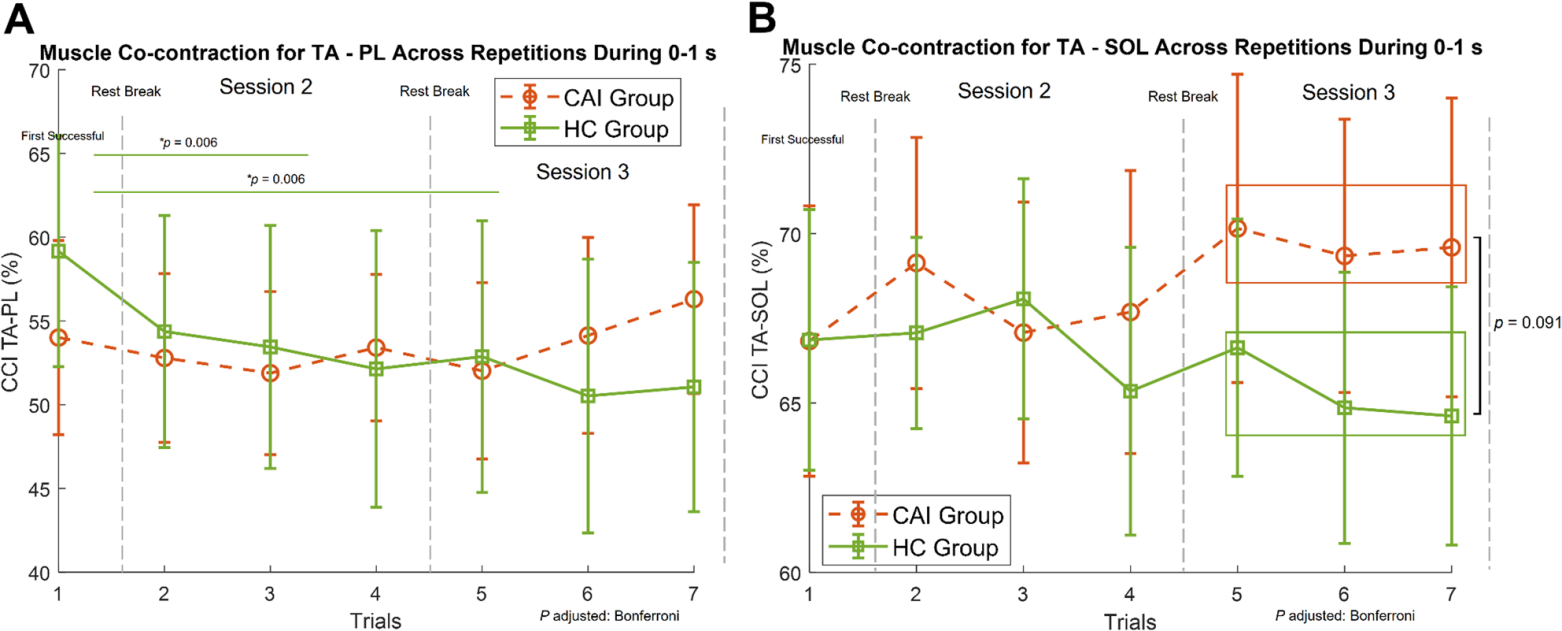
**A** Mean muscle co-contraction index (CCI) for Tibialis Anterior (TA), Peroneus Longus (PL) muscles (± 95% CI) during 1-s perturbation for CAI (dashed lines) and HC (solid lines) across seven successful trials in three sessions. Vertical dashed lines indicate session boundaries, each separated by a at least 5-minute seated rest. Trials 2–4 and 5–7 were averaged for statistical analysis. Post hoc comparisons revealed a significant decrease in muscle co-contraction as session progressed within HC group (*p* = 0.006, Bonferroni adjusted). **B** Mean muscle co-contraction index (CCI) for Tibialis Anterior (TA), Soleus (SOL) muscles (± 95% CI) during 1-s perturbation for CAI and HC across seven successful trials in three sessions. Vertical dashed lines and trial groupings are the same as in panel **A**

Both muscle pairs, TA-PL (F _(1.37, 59.04)_ = 4.48, *p* = 0.027) and TA-SOL (F _(2, 86)_ = 3.153, *p* = 0.048), exhibited significant interaction effects on muscle co-contraction (Table 2). Post hoc analysis showed that the HC group decreased TA-PL co-contraction in sessions 2 and 3 compared to session 1 (*p* = 0.006), indicating reduced muscle co-contraction as sessions progressed (Fig. 5A); whereas the CAI group showed no significant within-group changes in muscle co-contraction with repeated perturbations (*p* > 0.05). For the TA-SOL muscle pair, there was a nonsignificant trend toward decreased co-contraction in the HC group and increased co-contraction in the CAI group (*p* > 0.05). A significant negative correlation was found between CAIT scores and CCI TA-SOL (*r* = −0.551, *p* = 0.008), indicating individuals with poorer self-perceived ankle function exhibited greater TA-SOL muscle co-contraction. The correlation between CAIT and CCI TA-PL was not significant (*p* > 0.05).

**Table 2:** Mean and standard deviation (SD) for muscle co-contraction and cross-correlation between joint torques with MoS for CAI and HC among three sessions

	Time window	Group																		*P* values (Partial Eta Squared)					
		CAI (*n* = 22)									HC (*n* = 23)									Interaction		Group		Session	
		Session 1			Session 2			Session 3			Session 1			Session 2			Session 3			*p*	ηp^2^	*p*	ηp^2^	*p*	ηp^2^
MoS-Ankle Pairing	0–0.5 s	0.49	±	0.23	**†0.48**	**±**	**0.20**	**‡0.59**	**±**	**0.19**	0.57	±	0.28	0.62	±	0.21	0.59	±	0.21	0.136	0.045	0.161	0.045	0.334	0.025
MoS-Hip Pairing		**†0.29**	**±**	**0.20**	0.36	±	0.14	**‡0.49**	**±**	**0.21**	0.46	±	0.27	0.45	±	0.22	0.40	±	0.18	**0.002**	**0.135**	0.229	0.033	0.143	0.044
MoS-Ankle Pairing	0.5–1 s	0.59	**±**	0.28	0.60	**±**	0.18	**†0.57**	**±**	**0.21**	0.60	**±**	0.31	0.65	**±**	0.21	0.70	**±**	0.17	0.463	0.018	0.144	0.049	0.589	0.010
MoS-Hip Pairing		0.62	±	0.20	0.59	±	0.23	0.63	±	0.16	0.57	±	0.33	0.65	±	0.21	0.66	±	0.20	0.392	0.022	0.784	0.002	0.527	0.015
CCI TA - SOL	0–1 s	66.83	±	8.99	67.97	**±**	7.86	69.70	±	9.14	66.86	**±**	8.70	66.83	**±**	6.30	65.37	**±**	7.58	**0.048**	**0.068**	0.414	0.016	0.720	0.008
CCI TA - PL	0–1 s	54.01	±	12.17	52.69	±	10.23	54.15	±	11.46	**‡59.16**	**±**	**15.58**	53.32	±	15.89	51.49	±	17.18	**0.027**	**0.094**	0.792	0.002	**0.016**	**0.109**

†Denotes significant difference between CAI the HC at the session(*p* < 0.05). Bonferroni adjusted for multiple comparisons

‡Denotes significant within-group difference across three sessions (*p* < 0.05). Bonferroni adjusted for multiple comparisons

Effect Size was estimated by Partial Eta squared (ηp^2^). *CCI* Co-Contraction Index. *TA* Tibialis Anterior. *PL* Peroneus Longus. *SOL* Soleus. MoS-Joint Paring: absolute values of cross-correlation at zero-lag between Margin of Stability and joint torques

## Discussion

This study examined differences in adaptive changes in ankle muscle co-contraction and postural control between CAI and HC groups during balance practice using repetitive perturbation. Distinct patterns in muscle co-contraction for TA-PL and TA-SOL were observed between groups: the HC group showed a decrease in co-contraction, while no significant changes were found in the CAI group, partially supporting our hypothesis. CAI individuals with poorer self-perceived ankle function exhibited greater TA-SOL muscle co-contraction across practice. Practice improved postural control in the CAI group by decreasing the margin of stability and increasing MoS-Hip torque coordination. Additionally, individuals with CAI exhibited decreased hip and ankle torque across repeated perturbations, whereas no significant changes were observed in the HC group.

The current findings suggest different neuromuscular control strategies were adopted by CAI and HC groups since significant interaction effect on muscle co-contraction for TA-PL and TA-SOL were observed. In HC participants, a significant decrease in TA-PL co-contraction was observed, consistent with previous studies reporting reduced TA-gastrocnemius co-contraction across repeated anteroposterior perturbation trials [33], and decreased knee muscle co-contraction during repeated belt accelerations in walking [32]. For TA-SOL, a non-significant trend toward decreased co-contraction was observed in the HC group, likely because the mediolateral perturbations primarily targeted the TA-PL muscle pair. The decrease in co-contraction in our HC group represents reduced antagonist activation, which indicates improved energy efficiency and greater flexibility in balance recovery [28, 33]. No significant adaptive changes in co-contraction control were observed in the CAI group across repeated perturbations. The absence of a significant group main effect (*p* = 0.792) on TA-PL co-contraction between CAI and HC aligns with previous studies reporting no differences during single-leg landing tasks between CAI and HC [36, 37]. Nevertheless, these findings are in contrast to from other studies showing either increased [38] or decreased [39] TA-PL co-contraction in CAI individuals compared to healthy controls. Moreover, the greater TA-PL co-contraction in CAI than the controls during eyes-closed single-leg balance [35] indicates that co-contraction patterns might be task-dependant. Building on these studies, the current findings supply evidence of unchanged co-contraction patterns in CAI individuals across mediolateral repeated perturbations, suggesting a reduced adaptive capacity.

The predictability of repetitive perturbation characteristics including timing, magnitude, and direction modulates both anticipatory [56] and compensatory postural adjustments (APAs and CPAs) [57], through sensory information via visual, auditory, and tactile perceptions. For example, predictability of perturbations induces higher and earlier muscle response due to APAs [58], and has been shown to reduce muscle responses associated with CPAs [59]. Deficits in proprioception in people with CAI have been reported [60]; however, because proprioception tends to be a less efficient sensory resource than vision and audition for accurately predicting perturbations [61], differences in proprioception between populations seem to negligibly influence practice adaptation. Future studies should also test how CAI population adapt perturbation with different timing, magnitude and direction.

The unchanged co-contraction indicates that individuals with CAI, who exhibit impaired afferent input, may compensate by relying more heavily on conscious control [62]. Given that lower CAIT scores reflect greater perceived instability, greater pain and injury-related fear [63], the observed association between poorer self-perceived ankle function and higher TA-SOL co-contraction across practice implies a functional compensation for increased talocrural joint laxity for individuals with CAI [25, 34], but excessive co-contraction is maladaptive and dysfunctional, with greater energy expenditure [27] and impairing postural control [28]. Besides functional compensations and psychological factors [5], personal profile, such as injury history, physical attributes, and anatomical variations [64, 65], could further shape co-contraction responses, making this outcome highly multifactorial. Future study is warranted to investigate whether their co-contraction patterns contribute to future ankle sprain risk in CAI populations.

The distance between XCoM and BoS decreased with repetition at 0.18 s, when voluntary postural control began in response to perturbation, which typically starts after 0.15 s [66]. This suggests an enhanced feedforward postural control with practice in both groups, aligning with previous studies showing improved reactive balance control following acute exposure to repeated perturbations [33, 67, 68], and long-term perturbation-based balance training [69, 70]. With repeated perturbations the CAI group reduced MoS during the platform’s medial-to-lateral transition at 0.25 s, indicating a trainable postural response with improved effectiveness in postural recovery. The improvement in postural control in the CAI group is likely due to better coordination between MoS and hip joint torque. The interaction effect highlights a different practice effect on MoS-Hip torque pairing between groups. While the HC group exhibited reduced muscle co-contraction related to an improved muscular efficiency, the CAI group’s adaptation relied more on increased hip contribution, with no change in ankle muscle co-contraction.

Previous research has shown that individuals with ankle sprains [71, 72] and CAI [73] often adopt a hip-dominant strategy in the sagittal plane, characterised by increased hip extension torque and power during landing and jumping [73]. These strategies likely compensate for deafferented ankle joints to maintain postural control. Although balance training improves balance, self-perceived function and stability [74], it remains unclear whether the ankle contributions to balance are effectively enhanced. Interventions that modify somatosensory activation in the lower limb or encourage muscle activation through neural pathways to address arthrogenic muscle inhibition may help alleviate persistent symptoms [75, 76]. Based on the strategy of HCs in this study, effective hip, ankle strategy and less muscle co-contraction should be encouraged. To our knowledge, this is the first study in CAI population applying precise translational perturbations at the level of BoS as remedy. The perturbation development was built on traditional balance training methods such as single leg stance on wobble board tasks but provided a controllable perturbation across participants. Translational perturbations during standing were selected as a safer alternative to inverted or trapdoor perturbations [36, 77–79], with both mediolateral directions eliciting muscle co-contraction which also facilitates future investigations during walking, running, and the practical translation of balance training. Rehabilitation should focus on balance training that optimises whole body strategies, through approaches such as biofeedback [80, 81] or functional electrical stimulation. Developing clinically practical tools for assessing effective lower limb balance strategy might be helpful. Nevertheless, the absence of hip muscle EMG in this study limits our understanding of neuromuscular adaptations at the hip, warranting further research. This study indicates promises of ankle adaptations exercise, while their contribution to overall balance and injury prevention needs further investigations.

Repeated perturbations induced significant reductions in ankle and hip torque following practice in the CAI, but not in the HC group. This reduction may reflect improved feedforward control or a decrease in task demand. It also highlights a pathological characteristic of CAI, where individuals initially exert excessive torque for unfamiliar tasks. The excessive torque can tilt the foot onto the edge of the BoS, saturating the effective stabilising capacity [82], and potentially causing ankle sprains [83]. This overestimated torque might result from diminished somatosensation due to damage to ligaments, articular receptors, and muscle spindles [84], which are essential for accurately detecting force and velocity during movement [85]. Consequently, the central nervous system relies more on feedforward control, with torque levels adjusting through the practice. The reduction in torque reflects decreased task demand and suggests that torque might serve as a biomechanical marker to guide adjustments in training difficulty or postural demands. With advancements in wearable sensors (Inertial Measurement Units) and statistical models for estimating joint torques [86], CoM [87]and CoP [88], objective monitoring is now feasible in clinical settings.

This study has several limitations. The EMG for each muscle was normalised to the maximum value recorded across all successful perturbation trials, which limits the ability to compare amplitudes between groups. However, this method reduces variation across individuals and sessions [89]. As our primary aim was on adaptive patterns across sessions rather than co-contraction values between groups, the impact on interpretation should be minimal. Perturbations were delivered at a fixed 7-second interval, introducing predictability that may have influenced responses. Different intervals could influence responses to perturbations [90]. Lastly, our investigation focused on acute adaptations to repeated perturbations, which may not allow sufficient time for motor learning. Future longitudinal or retrospective studies are needed to clarify the development of CAI, by recording sprain history, sprain incidence, sprain/giving-way frequency, and personal profile, linking lab-based performance to real-world injury risk.

## Conclusion

Repetitive mediolateral perturbations induced different adaptive changes in muscle co-contraction in individuals with CAI compared to healthy controls. In the CAI, co-contraction remained unchanged in TA-PL and non-significantly increased for TA-SOL, whilst for healthy controls, TA-PL co-contraction decreased, but remained unchanged for TA-SOL. Repeated perturbations improved postural control in the CAI group by reducing the margin of stability and enhancing MoS-Hip coordination, indicating a trainable response with improved effective postural recovery with greater hip contribution. However, the improved ankle neuromuscular efficiency was evident in the healthy control group but was not observed for CAI, suggesting an impaired neuromuscular control adaptability to practice and changing postural demands. Rehabilitation for CAI should prioritise a whole-body movement coordination not ankle focused balance training approach since the ankle joint appears refractory to change in CAI individuals. Future longitudinal or retrospective studies are needed to clarify the development of CAI, by recording sprain history, sprain incidence, and personal profile, linking lab-based performance to real-world injury risk.

## Supplementary Information

Supplementary Material 1.

## Data Availability

The datasets analysed during the current study are available from the corresponding author (XX) on reasonable request.

## Abbreviations

CAI: Chronic ankle instability
MoS: Margin of stability
XCoM: Extrapolated centre of mass
BoS: Base of support
TA: Tibialis Anterior
PL: Peroneus Longus
HC: Healthy control
SOL: Soleus
RMS: Root mean square
EMG: Electromyography
CCI: Co-contraction index
