# Supplementary material for: Distinct patterns in neuromuscular adaptation to repeated perturbations in chronic ankle instability
Source: J Neuroeng Rehabil. 2025 Dec 12;23:21. doi: 10.1186/s12984-025-01838-y (PMC12817530; doi:10.1186/s12984-025-01838-y)
Supplement: Supplementary file 1 — Supplementary Material 1. [file 12984_2025_1838_MOESM1_ESM.docx]

**Supplementary**


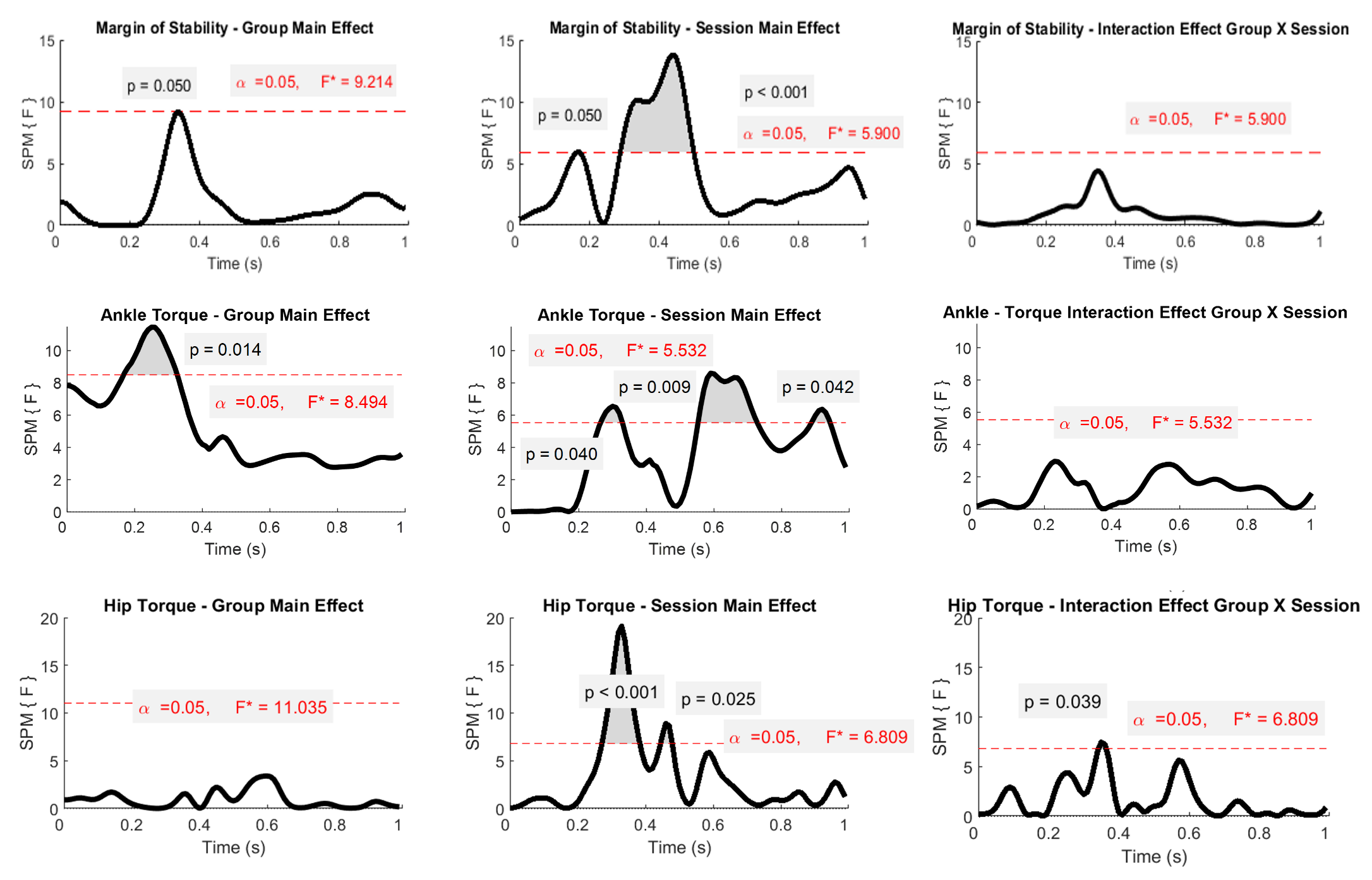


*Figure S1. Two-way repeated measures ANOVA results using Statistical Parametric Mapping (SPM) for Margin of Stability, ankle torque, and hip torque during the 1-second perturbation, analysing group, session, and interaction effects. The x-axis represents time (0–1 s), with the F-statistic plotted over time. The significance threshold (α = 0.05) is indicated by the red dashed line, and F* critical values for each analysis are highlighted.*
